# Effect of Low Versus High-Heeled Footwear on Spinopelvic Alignment at Different Phases of Menstrual Cycle in Young Adult Women: A Biopsychosocial Perspective

**DOI:** 10.3389/fpsyg.2021.792446

**Published:** 2021-11-24

**Authors:** Manal A. El-Shafei, Amel M. Yousef, Hamada A. Hamada, Mohamed F. Mohamed, Abdullah M. Al-Shenqiti, Ghada M. R. Koura, Guillermo F. López Sánchez

**Affiliations:** ^1^Department of Physical Therapy for Women’s Health, Faculty of Physical Therapy, Cairo University, Giza, Egypt; ^2^Department of Biomechanics, Faculty of Physical Therapy, Cairo University, Giza, Egypt; ^3^Consultant of Obstetrics and Gynecology, Om El Masryeen Hospital, Giza, Egypt; ^4^Faculty of Medical Rehabilitation Sciences, Taibah University, Medina, Saudi Arabia; ^5^Department of Medical Rehabilitation, Faculty of Applied Medical Sciences, King Khalid University, Abha, Saudi Arabia; ^6^Department of Physical Therapy for Musculoskeletal Disorder and Its Surgery, Faculty of Physical Therapy, Cairo University, Giza, Egypt; ^7^Faculty of Health, Education, Medicine and Social Care, School of Medicine, Vision and Eye Research Institute, Anglia Ruskin University, Cambridge, United Kingdom

**Keywords:** high heels, spinopelvic alignment, menstrual cycle, footwear, bio-psychosocial approach

## Abstract

High-heeled shoes adversely affect spinal curvature, increase the risk of low back pain, and disturb the normal gait pattern. The purpose of this study was to examine, from a biopsychosocial point of view, the combined effect of wearing two different heel heights and of hormonal oscillation throughout different phases of the menstrual cycle on spinopelvic alignment. Notably, 70 females with an average age of 20.42 ± 1.51 years participated in this study, wearing each female two different heel heights as follows: low (2.5 cm) and high (7 cm). Spinopelvic alignment was evaluated by rasterstereography formetric 3D analysis during early follicular, ovulatory, and mid-luteal phases of the menstrual cycle. Statistical analysis showed that there was no significant difference (*p* > 0.05) on spinopelvic alignment [kyphotic angle (KA), trunk inclination (TI), and pelvic inclination] between wearing low- or high-heeled shoes during early follicular, ovulatory, and mid-luteal phases of the menstrual cycle. Considering that high-heeled shoes are traditionally associated with femininity, body image, beauty, and charm, this research has important biopsychosocial implications that should be explored in detail in future studies.

## Introduction

High heels adversely affect the kinematics and kinetics of body structures from toes to the spine (Barnish and Barnish, 2016). Frequent wearing of high heels can cause long-lasting changes of body alignment in both adolescents and young adults, in addition to malposition of the spinal curvature and the lower limbs. Almost two-thirds of experienced high-heeled footwear users suffer from lumbar back pain (Weitkunat et al., 2016). Postural disorders caused by high-heeled shoes not only harm the musculoskeletal system but also disturb occupational health and activities of daily living (Karimi et al., 2016).

The menstrual cycle is characterized by oscillations of sex hormones, mainly estradiol and progesterone, in fertile females (Neill et al., 2009). Circulating estrogen level rises preovulatory and postovulatory while progesterone is confined to the postovulatory phase of the cycle as it is secreted by the corpus luteum (Reed and Carr, 2018). As estrogen and progesterone receptors are present in bone, ligaments, skeletal muscle, and the nervous system, it has been suggested that the hormonal level changes throughout different menstrual phases may affect the structure and function of these tissues, increasing the risk of injuries and also affecting female physical performance (Casey et al., 2014).

Although several studies examined the effect of high heels on posture and gait (Betsch et al., 2011; Dai et al., 2015; Lee et al., 2015), definitive findings have not yet been reported. Also, no previous studies have analyzed the combined effect of different heel heights and hormonal oscillation throughout different menstrual phases on spinal and pelvic alignment.

For this reason, this study aimed to explore, from a biopsychosocial point of view, the combined effect of two different heel heights and hormonal oscillation throughout different menstrual cycle phases (i.e., early follicular, ovulation, and mid-luteal), in young adult women, on thoracic kyphosis, TI, and pelvic inclination. We hypothesized that spinopelvic alignment would be affected by the combined effect of wearing different heel heights and hormonal fluctuations that occurred across the menstrual cycle, with important biopsychosocial implications.

## Materials and Methods

### Design

This study was cross-sectional with a repeated measures design.

### Participants

Notably, 70 female students were recruited through a flyer distributed at the Faculty of Physical Therapy of Cairo University, in Egypt [age: 20.42 ± 1.51 years; body mass: 61.17 ± 8.68 kg; height: 1.63 ± 0.06 cm; and body mass index (BMI): 22.70 ± 2.38 kg/m^2^]. The written informed consent was given to each participant after clarifying the aim of this study to them and their right of withdrawing from this study at any time. All participants signed the informed consent. Ethical approval was obtained from the Institutional Review Board of the Faculty of Physical Therapy of Cairo University before initiating this study (No. P.T.REC/012/001884). This trial was prospectively registered at clinicaltrials.gov: (NCT03688750). This study was conducted between October 2018 and May 2019.

### Eligibility Criteria

To be included in this study, the participants should have a regular menstrual cycle, an age from 19 to 25 years old, and a BMI from 18 to 25 kg/m^2^. Also, they were chosen to be non-experienced high-heeled users. Participants having an irregular menstrual cycle, spinal or foot deformities, or leg length inequality were excluded from this study. Also, habitual high-heel users were not included in this study to exclude adaptable changes of frequent use of high heels on the spine and foot (Farrag and Elsayed, 2016).

### Assessment

Demographic data of age, weight, height, and BMI of participants were obtained. Each female was asked to fill a self-administered questionnaire at the beginning of the study to collect data about their menstrual history, including menarche age, frequency (average 28 days), duration of menstruation (average 3–5 days), amount of menstrual flow (changing 2–3 pads per day) (Reed and Carr, 2018), and presence or absence of dysmenorrhea.

All participants were asked to record their menstrual cycle of the last 3 months by using a calendar, and the average cycle length was calculated to determine the time of each test. The measurements were performed during the early follicular (2nd or 3rd day), during ovulation (11–13 days), and mid-luteal phase (21–23 days). Urinary luteinizing hormone strip test (Shanghai International GmbH, Hamburg, Germany) was used for more accurate detection of the ovulatory phase. It was recommended to be used on the 11th and 12th day of the menstrual cycle when the positive result was obtained; the testing procedure was performed at the ovulatory phase (Hsiu-Wei et al., 2017).

### Shoe Condition

**FIGURE 1:**
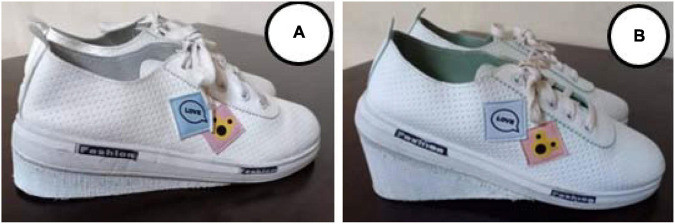
**(A)** Low-heeled shoes (2.5 cm) and **(B)** high-heeled shoes (7 cm).

Sport shoes with rubber wedged heels and two different heel heights (low 2.5 cm and high 7 cm) were used in this study (Figure 1).

### Rasterstereography

**FIGURE 2:**
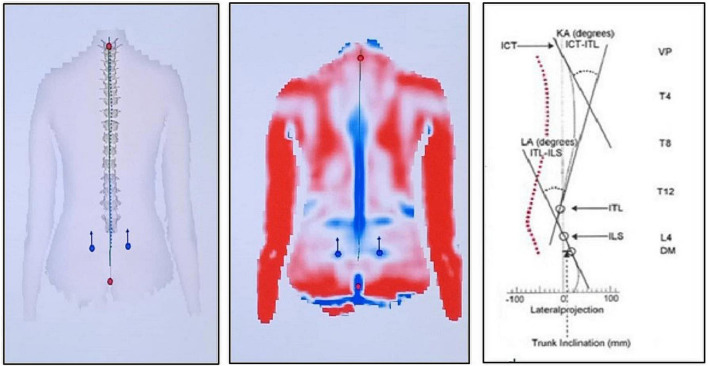
Illustrations of the shape of the spine and pelvis obtained from the DIERS formetric 3D system. The right image illustrates the sagittal profile of kyphotic angle (KA), lordotic angle (LA), trunk inclination (TI), cervicothoracic inflection point (ICT), thoracolumbar inflection point (ITL), and lumbosacral inflection point (ILS) (Schröder et al., 2019). The middle image illustrates the back surface reconstruction with red regions (convex curvature), blue regions (concave curvature), red dots referring to the vertebra prominence (VP), and blue dots referring to right and left lumbar dimples (DR and DL). The left image illustrates the frontal plane spine shape.

Spinopelvic alignment was examined using the three-dimensional rasterstereography Formetric II system (Diers International GmbH, Schlangenbad, Germany). It is a 3D analyzing method of back shape, with no radiation exposure. The patient can be studied automatically in a free-standing posture. In general, the analysis must refer to a proper coordinating system. In the case of rasterstereography, this is achieved analytically by referring to the so-called fixed-body coordinate system, which is fixed to C7 vertebra prominence and the midpoint between right dimple (DR) and left dimple (DL) (Figure 2). There are parallel light lines projected on the back surface and detected using a digital camera. This process only takes 0.04 s without any contact. Previous studies concluded that it has higher accuracy than radiograph, and it was a reliable method for 3D spinal alignment assessment in the sagittal plane (Drerup, 2014; Schröder et al., 2019).

### Outcome Measure

The selected parameters for this study were as follows:

- Kyphotic angle (KA): The angle lies between the upper inflection point nearby vertebral prominence (VP) and the thoracic-lumbar inflection point (ITL).
- TI: The angle lies between the gravity line and the line between VP anatomical landmarks and mid point between lumbar dimples (DM) (intermediate point between lumbar dimples). The angle is positive with VP anterior to DM (typical in leaning forward) and negative with VP posterior to DM (leaning backward).
- Pelvic inclination: This is calculated as the mean torsion of the DL and the DR, as explained by the instrument user guide (Figure 2).

There were no changes to trial outcomes after the trial commenced.

### Procedure

The participants were assigned to one group and randomly allocated to one of the two different shoe heel heights [low (2.5 cm) and high (7 cm)] through a computer-generated table of random numbers using Statistical Package for Social Sciences (SPSS) version 25 (SPSS, Inc., Chicago, IL, United States). Sequentially ordered cards were included in impermeable sealed envelopes and were opened by an assistant researcher blinded to the procedures, who allocated the participants to their shoes. The hair of females was bound up (cap, hair clips, or hair bands) to expose the neck and C7 vertebra prominence. Participant’s trunk was exposed till gluteal cleft. Rings, watches, and necklaces were removed to avoid any reflections. Each female stood in a relaxed position with a distance of nearly two meters from the system, and the position of the feet was marked on the floor to ensure that they stood in the same position in all trials.

Each female was asked to keep the head in the neutral position and breathe normally and then stop breathing for a few seconds while the image was captured. Full back 3D analysis was performed four times, and the mean value for each parameter was recorded. These steps were repeated for low- and high-heeled shoes, and approximately 5 min rest was given between measurements. These measurements were performed during different menstrual phases (i.e., early follicular, ovulatory, and mid-luteal phases).

### Sample Size Estimation

Before beginning the testing procedures, a pilot study was conducted with 10 participants to identify the proper sample size. Test size estimation was performed preceding the investigation utilizing G^∗^POWER statistical software (version 3.1.9.2; Franz Faul, Universitat Kiel, Germany) (*F*-tests – MANOVA: repeated measures, within factors, α = 0.05, β = 0.2, power = 80%, and partial η^2^ = 0.045, and effect size = 0.217), and it indicated that the proper sample size for this study was *N* = 56. We collected a larger number than the calculated sample size to overcome the possibility of missing participants.

### Statistical Analysis

Reported data were analyzed using the SPSS version 25, with an intention-to-treat analysis. Descriptive statistics, including mean ± SD, were quantified for all variables. This study involved two independent variables. The first independent variable was the sports shoes with different heel heights (within-subject factor) with two levels as follows: low (2.5 cm) and high (7 cm). The second independent variable was the phases of the menstrual cycle (within-subject factor) with three levels as follows: early follicular, ovulatory, and mid-luteal phases. The three dependent variables were as follows: KA, TI, and pelvic inclination. Two-way repeated measures MANOVA was applied to the dataset among the two heel heights during the three phases of the menstrual cycle. The alpha level was set at 0.05.

## Results

**FIGURE 3:**
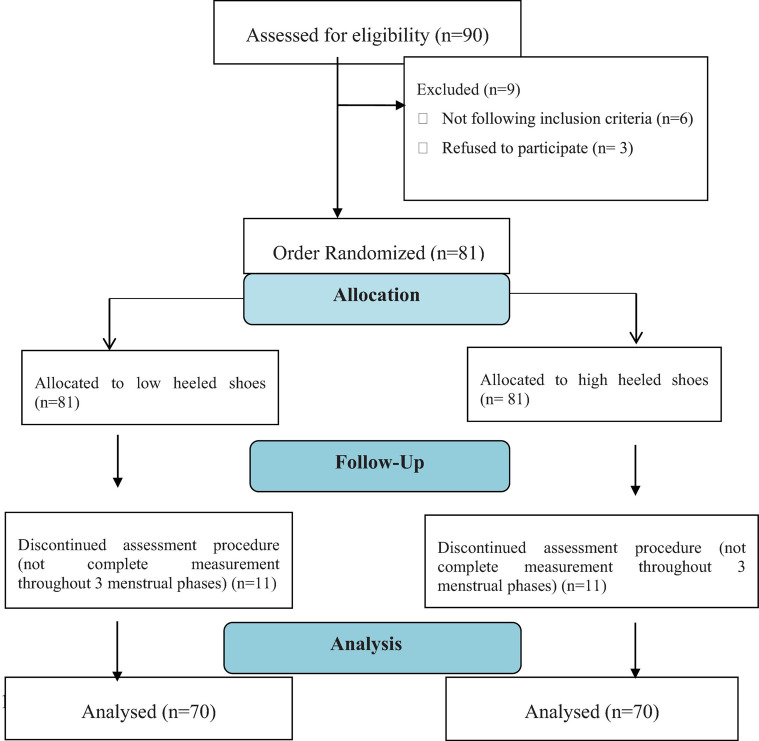
Flow diagram of this study.

Of the initial 90 participants, six of them did not follow the inclusion criteria, as they had irregular menstrual cycles or spinal deformities, which would affect the testing procedure and its results. Other five participants performed the assessment procedure in only one phase of menstruation. Other six participants performed the assessment procedure during two phases of menstruation but did not complete the last measurement. The other three participants refused to participate in this study due to personal reasons. Therefore, only 70 participants were included and analyzed (Figure 3).

Two-way repeated measures MANOVA for outcome measures indicated statistically non-significant effects for phases of menstrual cycle (*F* = 1.3, *p* = 0.34, and partial η^2^ = 0.08), sport shoes with different heel heights (*F* = 0.98, *p* = 0.54, and partial η^2^ = 0.17), and phases of menstrual cycle by sport shoes with different heel heights (*F* = 1.19, *p* = 0.33, and partial η^2^ = 0.21). Bonferroni multiple comparison tests (*post hoc* tests) indicated that there was no significant difference (*p* > 0.05) in KA, TI, and pelvic inclination among phases of the menstrual cycle at different heel heights. Also, there was no significant difference (*p* > 0.05) in KA, TI, and pelvic inclination between sports shoes with different heel heights at the three phases of the menstrual cycle (Table 1).

**TABLE 1:** Descriptive statistics for all variables (i.e., kyphotic angle, trunk inclination, and pelvic inclination) at early follicular, ovulatory, and mid-luteal phases.

	**Early follicular (phase I)**		**Ovulatory (phase II)**		**Mid-luteal (phase III)**	
**Variables**	**Low heel**	**High heel**	**Low heel**	**High heel**	**Low heel**	**High heel**
Kyphotic angle (degrees)	49.27 ± 9.11	48.79 ± 9.49	50.41 ± 9.23	49.13 ± 10.02	49.53 ± 8.63	49.83 ± 8.77
Trunk inclination (mm)	0.55 ± 3.18	0.67 ± 3.21	0.64 ± 3.07	0.77 ± 3.06	0.79 ± 3.75	0.86 ± 3.52
Pelvic inclination (degrees)	24.86 ± 8.87	24.37 ± 8.65	24.27 ± 8.53	24.79 ± 8.43	24.78 ± 8.80	25.21 ± 8.16

*Bonferroni multiple comparison tests (post hoc tests) for the all dependent variables between sport shoes with different heel heights at the three phases of menstrual cycle*						

**Variables**	**Low heel vs. High heel**					
	**Early follicular (phase I)**		**Ovulatory (phase II)**		**Mid-luteal (phase III)**

Kyphotic angle (degrees)	*p* = 0.99		*p* = 0.176		*p* = 0.99
	*d* = 0.052		*d* = 0.14		*d* = 0.035
Trunk inclination (mm)	*p* = 0.99		*p* = 0.99		*p* = 0.99
	*d* = 0.035		*d* = 0.041		*d* = 0.017
Pelvic inclination (degrees)	*p* = 0.371		*p* = 0.99		*p* = 0.99
	*d* = 0.055		*d* = 0.06		*d* = 0.048

*Bonferroni multiple comparison tests (post hoc tests) for the all dependent variables among the three phases of menstrual cycle at both sport shoes with different heel heights*						

**Variables**	**Phase I vs. Phase II**		**Phase I vs. Phase III**		**Phase II vs. Phase III**	
	**Low heel**	**High heel**	**Low heel**	**High heel**	**Low heel**	**High heel**

Kyphotic angle (degrees)	*p* = 0.280	*p* = 0.99	*p* = 0.99	*p* = 0.425	*p* = 0.561	*p* = 0.99
	*d* = 0.12	*d* = 0.03	*d* = 0.027	*d* = 0.11	*d* = 0.095	*d* = 0.07
Trunk inclination (mm)	*p* = 0.99	*p* = 0.99	*p* = 0.99	*p* = 0.99	*p* = 0.99	*p* = 0.99
	*d* = 0.027	*d* = 0.031	*d* = 0.074	*d* = 0.059	*d* = 0.048	*d* = 0.029
Pelvic inclination (degrees)	*p* = 0.99	*p* = 0.99	*p* = 0.99	*p* = 0.483	*p* = 0.836	*p* = 0.99
	*d* = 0.066	*d* = 0.048	*d* = 0.01	*d* = 0.097	*d* = 0.059	*d* = 0.05

*Significant at *p* < 0.05; *p*, *p*-value; *d*, Cohen’s *d*; Phase I, early follicular; Phase II, ovulatory; Phase III, mid-luteal; vs., versus.*

## Discussion

The use of high-heeled shoes leads to several postural changes, disturbs the balance, and causes joint injuries (Karimi et al., 2016). This study aimed to examine the combined effect of low and high-heeled shoes and hormonal oscillation throughout all menstrual cycle phases (i.e., early follicular, ovulatory, and mid-luteal phases) on spinopelvic alignment (i.e., KA, TI, and pelvic inclination). The findings of this study showed that there were no significant changes of KA, TI, and pelvic inclination while wearing low- (2.5 cm) and high-heeled shoes (7 cm) during early follicular, ovulatory, and mid-luteal phases.

The current results are in agreement with the previous study of Elsayed et al. (2018), who examined the impact of different heel heights on spinal posture and muscle activity in young adult women and found that there was no change in spinopelvic parameters (i.e., KA, TI, and pelvic inclination). Regarding KA, the current results are supported by Schröder et al. (2019) who investigated the impact of wearing personalized high heels on the woman posture at different ages and found that KA remained unchanged in younger wearers. Also, it is supported by the previous study of Weitkunat et al. (2016), who investigated the influence of high-heeled footwear usage on the sagittal balance of the spine and the whole body (using whole body photograph) and found that there were no statistically significant changes of thoracic kyphosis and lumbar lordosis in high-heeled footwear. Furthermore, our findings are confirmed by Iunes et al. (2008), who evaluated the influence of high heels on body posture among adult women and found no changes in LA, KA, and pelvic tilt concerning the frequency of high heel usage and shoe types.

In contrast, Baaklini et al. (2017) revealed with their study that non-experienced women wearing high-heeled shoes have a lower angle of thoracic curvature than those wearing low-heeled shoes. Also, they reported that nevertheless of their experience in wearing footwear with heels, women wearing high heels revealed the lower minimum and maximum thoracic and lumbar curvature angles than those in bare feet. Also, Dai et al. (2015) reported that the increase of heel height leads to higher kyphotic and LAs in young females. At the same time, the gravity line shifted significantly anteriorly.

Regarding TI, the current result is supported by the previous studies of Elsayed et al. (2018) and Schröder et al. (2019), who revealed that TI remains unchanged in high-heeled shoe users. In contrast, our results disagree with Drzal-Grabiec and Snela (2013), who concluded that forward TI increased gradually with increased heel heights.

Regarding pelvic inclination, the current result is supported by the previous studies of Weitkunat et al. (2016) and Elsayed et al. (2018), who revealed that pelvic inclination remains unchanged in high-heeled shoe users. However, Michoński et al. (2019) concluded that vertical TI and pelvic inclination decreased due to mid-high–heeled shoes in young adult women.

The non-significant changes in spinopelvic parameters obtained from our study may be explained because the primary compensation would occur in the lower limb in non-habitual high-heeled shoe wearers, with knee flexion compensating initially the ankle plantar flexion. Later on and after knee muscles fatigue, pelvis, and trunk begin to compensate for the forward shift of the line of gravity. Moreover, kinematic chain compensations could primarily occur in the lower extremity and pelvis with no changes in spine curves (Betsch et al., 2011).

Regarding the impact of the different menstrual phases on spinopelvic alignment, the findings of this study revealed that there is no significant difference in KA, TI, and pelvic inclination during early follicular, ovulatory, and mid-luteal phases. Until now, we are aware of only one study in the literature that investigated hormonal oscillations of state anxiety, spinal structure, and postural stability through the menstrual cycle in active women (Kaya and Çelenay, 2016). Regarding spinal structure evaluation, they used the spinal mouse to detect the spinal process from C7 to S3, with the subjects in standing position and repeating the measurement throughout the different menstrual phases. Our results show similarities with their findings, as they found that there was no significant change in spinal posture throughout different menstrual cycles. It was concluded that spinal stability involved three components as follows: ligaments (passive system), muscles (active system), and neural control, which guards against spinal injury and also permits the desired movement (Panjabi, 1992). We will discuss the present results from this point of view.

It has been stated that hormonal fluctuations act on the central nervous system, causing changes in the activity of the autonomic nervous system. Furthermore, it has been reported that, during the menstrual cycle, the baroreflex regulation of autonomic functions induced by altering positions changed (Kaya and Çelenay, 2016). Hormonal oscillation during different menstrual phases adversely affects the structure of ligaments (the passive stabilizing system of the spine), reducing ligament tension and increasing the incidence of tear (Shakeri et al., 2013). Previous studies analyzed the correlation between hormonal fluctuations throughout the menstrual cycle and ligament laxity, but there were conflicting results (Vescovi, 2011; Herzberg et al., 2017).

The results of this study are confirmed by Vescovi (2011), who found that there was no correlation between acute hormonal fluctuation during menstrual phases and knee/ankle laxity. Also, it agrees with Park et al., 2009 who concluded that there were no significant changes in knee joint mechanics through the menstrual cycle (no phase influence). Furthermore, our results agree with those of Beynnon et al. (2005) and Hoffman et al. (2008), who did not find changes in knee laxity throughout the menstrual cycle. Conversely, Herzberg et al. (2017) found that the liability of anterior cruciate ligament (ACL) injury increased particularly during ovulation due to a high level of estradiol. Similarly, Lefevre et al. (2013) concluded that ACL tear in females occurs more frequently in the follicular and ovulatory phases than in the luteal phase.

Regarding the effect of hormonal fluctuation on muscle strength, the findings of this study are supported by Burgess et al. (2010), who could not find any changes in the mechanical properties of tendons. Also, Abt et al. (2007) found that all neuromuscular and biomechanical characteristics persisted constantly in spite of changes in circulating estrogen and progesterone level throughout the phases. Moreover, Montgomery and Shultz (2010) and Wild et al. (2013) reported that there was no relation between hormonal fluctuation and changes in muscle strength. Conversely, the current results disagree with those of Ronkainen et al. (2009) and van Geel et al. (2009), who reported that changes in muscle strength were associated with the alteration of the level of estradiol hormone.

This study has important strengths. At present, this is the first study that has investigated the combined effect of wearing different heel heights and of hormonal oscillation that occurred across different menstrual phases on spinal and pelvic alignment. Also, the methods of this study were valid and reliable. However, some limitations should be considered too in this study. It was conducted only on Egyptian female students, and the tests were carried out throughout only one menstrual cycle. Furthermore, hormonal profile analysis for estrogen and progesterone hormones was not performed to detect the exact timing of each menstrual phase. Therefore, similar studies are suggested to be carried out on samples from different countries. Finally, an evaluation lasting for more than one cycle, and with more accurate detection of the timing of each menstrual phase through hormonal analysis, is recommended to be performed in future studies, as it may provide different results.

## Conclusion

From a biopsychosocial point of view, there is no influence of wearing low- or high-heeled shoes on spinopelvic alignment (i.e., thoracic kyphosis, TI, and pelvic inclination) during early follicular, ovulatory, and mid-luteal phases of the menstrual cycle. Considering that high-heeled shoes are traditionally associated with femininity, body image, beauty, and charm, this research has important biopsychosocial implications that should be explored in detail in future studies.

## Data Availability Statement

The raw data supporting the conclusions of this article will be made available by the authors, without undue reservation.

## Ethics Statement

The studies involving human participants were reviewed and approved by the Institutional Review Board of the Faculty of Physical Therapy of Cairo University (No. P.T.REC/012/001884). The participants provided their written informed consent to participate in this study.

## Author Contributions

ME-S, AY, and HH contributed to the concept and design of the study, collected the data, and performed the statistical analysis and data interpretation. All authors collaborated in writing and critical revision of the study, and read and agreed to the published version of the manuscript.

## Conflict of Interest

The authors declare that the research was conducted in the absence of any commercial or financial relationships that could be construed as a potential conflict of interest.

## Publisher’s Note

All claims expressed in this article are solely those of the authors and do not necessarily represent those of their affiliated organizations, or those of the publisher, the editors and the reviewers. Any product that may be evaluated in this article, or claim that may be made by its manufacturer, is not guaranteed or endorsed by the publisher.
